# Comparison of size modulation and conventional standard automated perimetry with the 24-2 test protocol in glaucoma patients

**DOI:** 10.1038/srep25563

**Published:** 2016-05-05

**Authors:** Kazunori Hirasawa, Nobuyuki Shoji, Masayuki Kasahara, Kazuhiro Matsumura, Kimiya Shimizu

**Affiliations:** 1 Orthoptics and Visual Science, Department of Rehabilitation, School of Allied Health Sciences, Kitasato University, Kanagawa, Japan; 2 Department of Ophthalmology, School of Medicine, Kitasato University, Kanagawa, Japan

## Abstract

This prospective randomized study compared test results of size modulation standard automated perimetry (SM-SAP) performed with the Octopus 600 and conventional SAP (C-SAP) performed with the Humphrey Field Analyzer (HFA) in glaucoma patients. Eighty-eight eyes of 88 glaucoma patients underwent SM-SAP and C-SAP tests with the Octopus 600 24-2 Dynamic and HFA 24-2 SITA-Standard, respectively. Fovea threshold, mean defect, and square loss variance of SM-SAP were significantly correlated with the corresponding C-SAP indices (P < 0.001). The false-positive rate was slightly lower, and false-negative rate slightly higher, with SM-SAP than C-SAP (P = 0.002). Point-wise threshold values obtained with SM-SAP were moderately to strongly correlated with those obtained with C-SAP (P < 0.001). The correlation coefficients of the central zone were significantly lower than those of the middle to peripheral zone (P = 0.031). The size and depth of the visual field (VF) defect were smaller (P = 0.039) and greater (P = 0.043), respectively, on SM-SAP than on C-SAP. Although small differences were observed in VF sensitivity in the central zone, the defect size and depth and the reliability indices between SM-SAP and C-SAP, global indices of the two testing modalities were well correlated.

Conventional standard automated perimetry (SAP) has a constant stimulus size during the entire testing session. The test is performed by presenting stimuli produced with the projection light source in a dome-shaped bowl. By changing the light source and stimulus presentation plane, it is possible to obtain a wide stimulus dynamic range for determining visual sensitivity. Although SAP can theoretically be performed with a liquid crystal display (LCD) monitor, this is not usually done in the clinical setting because it is difficult to maintain a wide stimulus dynamic range, which is limited by the maximum intensity of the LCD monitor. On the other hand, perimetry measurements with a particular stimulus (e.g., pulsar perimetry^1^,^2^,^3^, motion displacement test^4^,^5^, flicker-defined form perimetry^6^,^7^, frequency doubling technology^8^,^9^, and high-pass resolution perimetry^10^,^11^,^12^) must be performed with a computer display because it is difficult to produce particular stimuli and present them in the stimulus plane with a projection light source.

The Octopus 600 perimeter (Haag-Streit, Koeniz, Switzerland), which is based on a thin film transistor LCD, was recently designed to perform both pulsar perimetry^1^,^2^ and SAP^13^. Because the LCD monitor has a limited maximum intensity, it is difficult to obtain the traditional stimulus dynamic range of SAP using only Goldmann stimulus size III^13^. To address this limitation of SAP performed with an LCD monitor, the Octopus 600 utilizes the novel technique of stimulus size modulation. With this technique, the stimulus size of high-intensity stimuli more than 10 dB is increased to maintain a stimulus intensity of 10 dB, and the size of low-intensity stimuli less than 24 dB is decreased to maintain an intensity of 24 dB^13^,^14^. This allows the spatial summation of the total light for each stimulus to remain constant across all stimuli. This technique has been previously validated in the clinical setting^13^.

Many studies have examined the variability and detection of visual field defects measured with SAP using Goldmann stimulus sizes I to VI^15^,^16^,^17^,^18^,^19^,^20^,^21^,^22^,^23^,^24^,^25^,^26^,^27^. These studies have demonstrated improved test–retest variability and higher detection sensitivity, both of which are dependent upon stimulus size^15^,^16^,^17^,^18^,^19^,^20^,^21^,^22^,^23^,^24^,^25^,^26^,^27^. However, few investigations have examined size modulation SAP (SM-SAP), in which stimulus size is varied during testing^13^. A previous study^13^ compared conventional SAP (C-SAP) performed with the Octopus 311 and SM-SAP performed with a prototype pulsar perimeter, both of which have the same maximum stimulus intensity and the same strategy of tendency-oriented perimetry.

Although the Octopus perimeter and the Humphrey Field Analyzer (HFA) have different maximum stimulus intensities and different measurement algorithms, these perimeters have been commonly used in a clinical setting. Therefore, it would be useful to understand the differences and similarities between SM-SAP results and C-SAP results obtained with the HFA (Carl Zeiss Meditec, Dublin, CA). The current study evaluated the characteristics of SM-SAP testing results obtained with the Octopus 600 and compared them with C-SAP testing results obtained with the HFA.

## Results

After two eyes of two patients were excluded due to the high false-positive (FP) rate in SM-SAP, 88 eyes of 88 glaucoma patients were analyzed in the study. Table 1 summarizes the subject demographic and ocular data.

**Figure 1:**
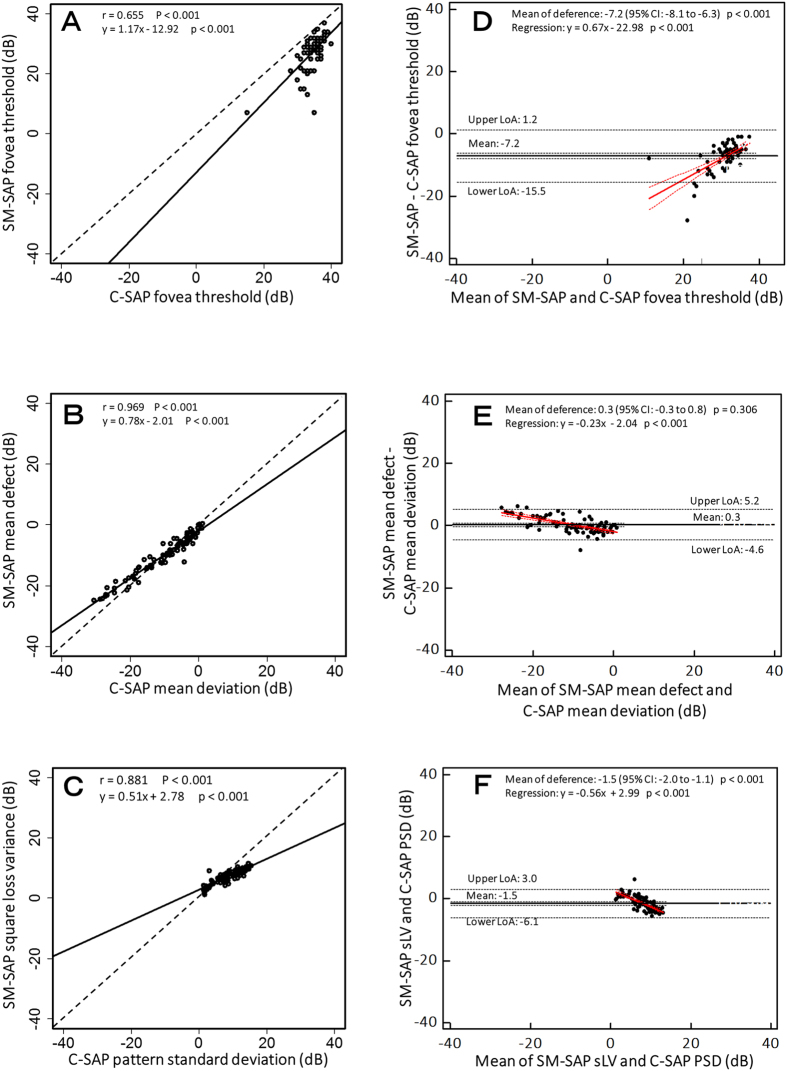
Correlations (**A**–**C**) and Bland–Altman analysis (**D**–**F**) between size modulation standard automated perimetry (SM-SAP) and conventional standard automated perimetry (C-SAP) global indices. Scatter plots show comparisons of the SM-SAP fovea threshold, mean defect, and square loss variance (sLV) and the C-SAP fovea threshold, mean deviation, and pattern standard deviation (PSD), respectively. The best-fit line equation, correlation coefficient (r), and P value are shown for each parameter comparison. The dashed line indicates the y = x line. Bland–Altman plots (**D**–**F**) show the mean difference and limits of agreement (LoA) as black solid and dashed lines, respectively. Red solid and dashed lines show the best-fit line and its 95% confidence interval (CI) line of proportional bias, respectively. The sign of the SM-SAP mean defect was reversed to correspond to the sign of the C-SAP value.

The correlation between the SM-SAP and C-SAP global indices (Fig. 1A–C) revealed that the SM-SAP and C-SAP fovea threshold measurements were moderately but significantly correlated (r = 0.655, P < 0.001). However, the SM-SAP mean defect and the C-SAP mean deviation parameters (r = 0.969, P < 0.001) and the SM-SAP square loss variance (sLV) and the C-SAP pattern standard deviation (PSD) parameters (r = 0.881, P < 0.001) were strongly correlated. Figure 1D–F shows the Bland–Altman analysis between the SM-SAP and C-SAP global indices. The mean difference between the SM-SAP and C-SAP fovea threshold was −7.2 dB (95% confidence interval [CI]: −8.1 to −6.3 dB, P < 0.001); the SM-SAP mean defect and C-SAP mean deviation was 0.3 dB (95% CI: −0.3 to 0.8 dB, P = 0.306), and the SM-SAP sLV and C-SAP PSD was −1.5 dB (95% CI: −2.0 to −1.1 dB, P < 0.001). A fixed bias was demonstrated between all indices but not between the SM-SAP mean defect and the C-SAP mean deviation, and a proportional bias was demonstrated between all SM-SAP and C-SAP global indices (P < 0.001). The upper and lower limits of agreement (LoA), best-fit line equation of proportional bias, and P value are shown in Fig. 1D–F.

**Figure 2:**
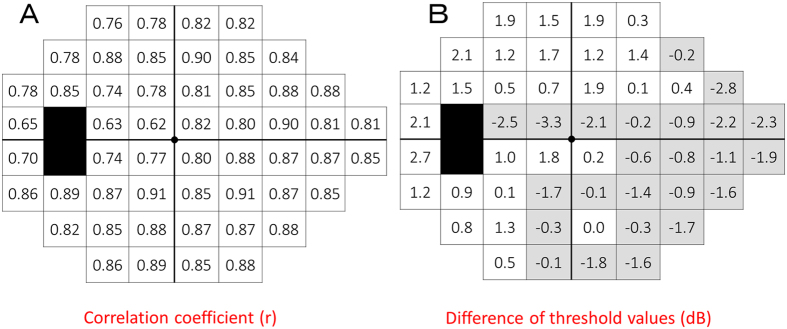
Comparison between size modulation standard automated perimetry (SM-SAP) and conventional standard automated perimetry (C-SAP) point-wise thresholds. Correlation coefficients (**A**) and differences between testing modalities in point-wise threshold values (**B**) are shown. The sign of SM-SAP comparison values was reversed to correspond to that of C-SAP values. The black area represents the blind spot. The grey area represents high SM-SAP thresholds compared with C-SAP thresholds.

**Figure 3:**
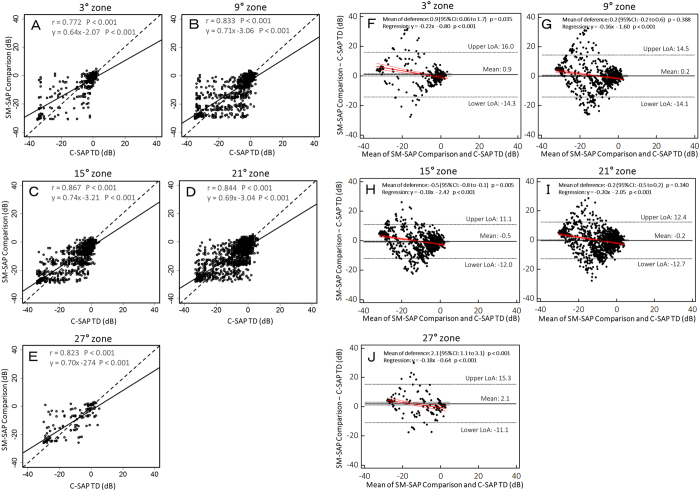
Scatter plots (**A**–**E**) and Bland–Altman plots (**F**–**J**) between size modulation standard automated perimetry (SM-SAP) and conventional standard automated perimetry (C-SAP) threshold values in each measurement zone. Measurement zones were macula-centered circles with eccentricities of 3°, 9°, 15°, 21°, and 27°, as labelled (see Fig. 5). The dashed line indicates the y = x line. The correlation coefficient (r) and P value are shown for each parameter comparison. Bland–Altman plots (F to J) show the mean difference and limits of agreement (LoA) as black solid and dashed lines, respectively. Red solid and dashed lines show the best-fit line and its 95% confidence interval (CI) line of proportional bias, respectively. The sign of the SM-SAP comparison values was reversed to correspond to that of the C-SAP values.

Correlation coefficients and differences in point-wise threshold values, calculated using the SM-SAP comparison values and the C-SAP total deviation (TD) values, are shown in Fig. 2. Figure 3A–J show the correlation and difference in the individual points from each zone as scatter plots and Bland–Altman plots, respectively. All test points were moderately to strongly correlated between testing modalities (all P < 0.001). Correlation coefficients for the central 3°, 9°, 15°, 21°, and 27° zones were 0.772, 0.833, 0.867, 0.844, and 0.823, respectively (all P < 0.001). The correlation coefficient for the 3° zone was significantly lower than those of the 15° (χ^2^ = 21.33, P < 0.001) and 21° (χ^2^ = 8.69, P = 0.032) zones. The correlation coefficients for the 9° (χ^2^ = 29.50, P < 0.001) and 21° (χ^2^ = 17.76, P < 0.001) zones were significantly lower than that of the 15° zone. These P values were corrected using the Bonferroni method. When differences in sensitivity were examined at each test point, sensitivities from the blind spot to the central region and to the superior and inferior nasal regions tended to be approximately 1 to 3 dB higher on SM-SAP than on C-SAP. The sensitivity in other regions tended to be approximately 1 to 3 dB higher on C-SAP than on SM-SAP. Figure 3F–J shows the difference in the individual points from each zone between SM-SAP and C-SAP as Bland–Altman plots. The mean difference between the SM-SAP and C-SAP thresholds in the 3° zone was 0.9 dB (95% CI: 0.06 to 1.7 dB, P = 0.035); 9° zone, 0.2 dB (95% CI: −0.2 to 0.9 dB, P = 0.388); 15° zone, 0.5 dB (95% CI: −0.8 to −0.1 dB, P < 0.001); 21° zone, −0.2 dB (95% CI: −0.5 to 0.2 dB, P = 0.340); and 27° zone, 2.1 dB (95% CI: 1.1 to 3.1 dB P < 0.001). A fixed bias was demonstrated in all zones except the 9° and 21° zones, and a proportional bias was demonstrated in all zones (all P < 0.001). The upper and lower LoA, best-fit line equation of the proportional bias, and P values are shown in Fig. 3F–J.

The visual field defect size and depth were examined in the 75 eyes of 75 subjects in which a pattern deviation value and its probability plot were calculated by C-SAP with the HFA. The visual field defect size was significantly 3 points smaller on SM-SAP than on C-SAP (P = 0.039, paired *t*-test), and the visual field defect depth was significantly 2 dB greater on SM-SAP than on C-SAP (P = 0.043, paired *t*-test). The test duration was 18.0% shorter on SM-SAP than on C-SAP (P < 0.001, paired *t*-test).

The reliability of the two testing modalities was examined and compared. The false-negative (FN) rate was only examined in 84 eyes of 84 subjects in which the FN rate was calculated by C-SAP with the HFA. The FP rate was slightly, but significantly, higher with C-SAP (median = 1.0%) than with SM-SAP (median = 0%, P = 0.002, Wilcoxon signed-rank test). However, the FN rate with SM-SAP (median = 7.1%) was significantly higher than with C-SAP (median = 1.5%, P < 0.001, Wilcoxon signed-rank test).

**Figure 4:**
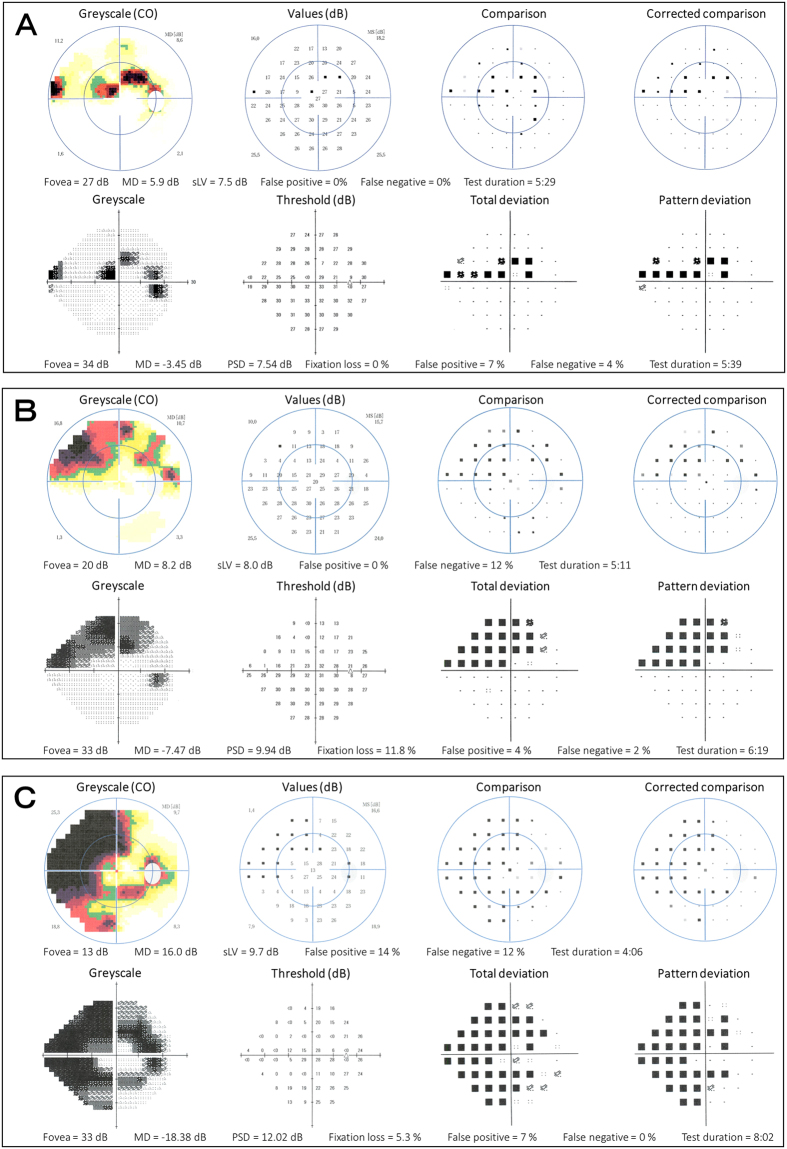
Representative size modulation standard automated perimetry (SM-SAP) and conventional standard automated perimetry (C-SAP) test results of three glaucoma patients with early (**A**), moderate (**B**), and severe (**C**) visual field defects. The SM-SAP and C-SAP test results are shown in the superior and inferior hemifields, respectively.

Comparisons of these parameters measured with SM-SAP and C-SAP are shown in Table 2. Representative test results of three glaucoma patients with early, moderate, and severe defects are shown in Fig. 4.

## Discussion

We found a moderate, but significant, correlation between fovea threshold measurements made using SM-SAP and C-SAP. The C-SAP foveal and peripheral thresholds obtained with the HFA were measured in different testing sessions. In contrast, the SM-SAP foveal and peripheral thresholds obtained with the Octopus 600 were measured during a single testing session. Fujimoto *et al.*^28^,^29^,^30^,^31^ showed that the thresholds of central test points were significantly higher when a smaller measurement area and a smaller number of test points were used. This phenomenon was thought to be associated with subject attention for the area of the presented stimulus. The previous study reported that the variability increased as the stimulus size decreased^17^,^22^,^25^,^26^. The low-intensity stimuli (>24 dB) were smaller in size on SM-SAP than on C-SAP. Therefore, smaller stimuli were presented during SM-SAP because nearly all subjects had a higher fovea threshold (Fig. 2). Even if the difference of approximately 4 dB due to the difference in maximum stimulus intensity between the HFA C-SAP and Octopus 600 SM-SAP was considered, the SM-SAP fovea threshold was approximately 3 dB lower than that obtained using C-SAP.

Point-wise and zone threshold measurements made with SM-SAP were strongly correlated with those measured with C-SAP, except near the fixation point. Previous studies report that the test–retest variability in regions of decreased sensitivity was higher than in regions of normal sensitivity in glaucoma patients^32^,^33^,^34^. Considering the threshold variability in the current study, we would expect correlation coefficients at each test point and in each zone to generally be high. The Octopus 600 fixation target is a crosshair with a visual angle of 2.7°, but the HFA fixation target is a small circle with a visual angle of approximately 1.1°. Previous studies have reported that fixation disturbances during visual field testing increase with increasing target size^35^,^36^, in glaucoma patients^37^, and with increasing magnitude of the visual field defect^38^. It has also been reported that slight fixation disturbances of approximately 2.9° can occur during reliable visual field testing^39^ and that fixation disturbances of <2.5° occur in up to 60% of reliable visual field tests^40^. Additionally, ganglion cells are most heavily concentrated in the macular area, and ganglion cell density decreases as eccentricity increases^41^. Therefore, cells in the macular area have more narrow cellular receptive fields and cells in the periphery have wider receptive fields. The weak correlation in the central region, compared with the moderate correlation in the middle to peripheral area, might have occurred because of fixation disturbances caused by size differences in the fixation target, ganglion cell distribution, and measured receptive field.

Regarding differences in threshold at each test point, thresholds from the blind spot to the central, superior nasal, and inferior nasal regions were approximately 1–3 dB higher when measured with SM-SAP than when measured with C-SAP. Thresholds in other regions were approximately 1–3 dB higher when measured with C-SAP than when measured with SM-SAP. A previous study reported that the threshold at each test point was approximately 1–3 dB higher with the Swedish interactive threshold algorithm (SITA)-Standard than with the Dynamic strategy in normal subjects^42^. Another study reported that local defects were deeper with the Dynamic strategy than with the SITA-Standard in the pre-perimetric and early stages of glaucoma, but that the reverse was true for patients in the moderate to severe disease stages^43^. The regions from the blind spot to the central, superior nasal, and inferior nasal regions are easily damaged in glaucoma patients. In the current study, these regions tended to have lower sensitivities on SM-SAP than on C-SAP (Fig. 2B).

The SM-SAP mean defect and sLV were strongly correlated with the corresponding C-SAP mean deviation and PSD, respectively. This is almost in agreement with a previous study^13^, but comparing our study results with the previous study’s results is difficult because of the use of different measurement conditions. Another previous study also reported that the Dynamic strategy gave lower estimates of localized loss than did the SITA strategy^43^, and we found similar results. These global indices were calculated using the deviation from age-corrected normal threshold data even though calculation details were slightly different between the HFA and Octopus 600 perimeters. Therefore, global indices of similar values would translate into a decreased SITA standard sensitivity. However, regions of 0 to 4 dB on C-SAP with the HFA were measured as 0 dB on SM-SAP with the Octopus 600 because of the difference in the maximum stimulus range. Therefore, there was a slight difference between PSD and sLV values in the current study.

Visual field defects were smaller and deeper on SM-SAP than on C-SAP. The normal limits of each test point with the SITA-Standard can be restricted to 9% to 29% at all probability levels, particularly in the middle to peripheral areas^44^. Previous studies reported that visual field defect size and depth on SITA testing were greater and smaller, respectively, than those detected with the conventional full threshold strategy^45^,^46^. It is possible that visual field defect size was affected by age-corrected normative data limit differences between the SAP devices. Although the C-SAP SITA-Standard strategy presents stimuli in intervals of 4–2 dB in both abnormal and normal regions^47^, the SM-SAP Dynamic strategy used in our study presents stimuli in intervals of 2 dB at high sensitivity and 10 dB at low sensitivity^48^. Therefore, the sensitivity in abnormal regions on SM-SAP tended to decrease more than on HFA C-SAP.

The test duration was approximately 18% shorter with SM-SAP than with C-SAP in the current study. A previous study reported that the C-SAP test duration with the SITA-Standard and the Dynamic strategies in normal and early glaucoma patients was the same, but that testing with the Dynamic strategy in moderate to severe glaucoma patients was approximately 6% to 17% shorter than with the SITA-Standard strategy^43^. The Dynamic and SITA-Standard strategies are typically time-saving strategies for presenting stimuli that account for the frequency-of-seeing curve^49^,^50^. The Dynamic strategy reduces the number of stimulus presentations by expanding the interval for depressed retinal sensitivity points with a more shallow slope of the frequency-of-seeing curve^51^. On the other hand, the SITA-Standard strategy presents stimuli in intervals of 4–2 dB for both abnormal and normal regions^47^. Differences in test duration for the SM-SAP Dynamic strategy and the C-SAP SITA-Standard strategy in the current study would be associated with differences in stimulus intensity interval.

Although the FP rate was higher with C-SAP than with SM-SAP, we found that the FN rate was higher with SM-SAP than with C-SAP. In traditional threshold perimetry, the FP and FN rates were estimated by adding extra stimulus presentations, called catch trials^52^. However, the SITA strategy uses a different method for estimating FP and is based on the reaction time to a 180- to 200-msec stimulus presented after the main testing stimuli^47^. The FP rate during C-SAP with the SITA-Standard strategy is calculated for all stimuli presented during the test. In contrast, the FP rate during SM-SAP testing with the Dynamic strategy is only based on the extra stimuli of catch trials. Therefore, it is thought that the FP rate of C-SAP is generally higher than that of SM-SAP. Although the SITA and Dynamic strategies both use only traditional catch trials to measure the FN rate, different stimulus presentation methods are used for each testing modality. The FN rate is measured with the SITA strategy using stimulus intensities that are 20 dB greater than the previously determined threshold at predetermined point locations with normal or almost normal sensitivity^53^. The FN rate of the Octopus 600 Dynamic algorithm used in the current study was measured using a maximum intensity stimulus (0 dB) at predetermined point locations with both normal and abnormal sensitivity. It is known that abnormal locations in glaucoma patients have a higher stimulus variability^32^,^33^,^34^,^54^. Specifically, the higher FN rate on SM-SAP was influenced by stimulus response variability in regions with abnormal sensitivity.

Octopus 600 SM-SAP presented some limitations for our study. First, there is considerable variability in the k value from one individual to another. Secondly, the spatial summation function is linear for some regions (Ricco’s Law), but the slope changes for larger targets (Piper’s Law)^55^. Third, the relationship between the size and luminance of the target is quite complex and varies as a function of stimulus intensity, background adaptation level, target size, and other parameters^56^. Finally, the relationship between size and intensity is not always linear^57^. These limitations should be considered in future studies.

In conclusion, SM-SAP performed with the LCD-based Octopus 600 perimeter had characteristic test results. The correlations between foveal measures made with C-SAP on the HFA and SM-SAP on the Octopus 600 were particularly weak. These parameters included fovea threshold, visual field defect characteristics (size and depth), and reliability indices. These characteristics of SM-SAP should be considered when evaluating SM-SAP results and comparing them with C-SAP results. However, these specific differences in the characteristic testing results likely reflect differences in device modalities rather than the effect of size moderation. Although further investigation is likely needed for threshold correlation of central areas, global indices with SM-SAP and C-SAP were generally highly consistent between the two perimetry methods.

## Methods

This prospective randomized study was reviewed and approved by the Kitasato University Hospital Ethics Committee (no. B14-129). All study conduct adhered to the tenets of the Declaration of Helsinki, and all study subjects provided written informed consent. This study was registered in the UMIN Clinical Trials Registry (http://www.umin.ac.jp/) under the unique trial number UMIN000016055 (date of registration: 12/25/2014).

### Study subjects

Glaucoma patients who visited the Kitasato University Hospital Glaucoma Service between January and May 2015 were recruited for enrollment if they had good central fixation and reliable HFA visual field measurements with the SITA standard 24-2 testing protocol. The HFA visual field testing results were considered reliable if the fixation loss was <20% and the FP rate was <15%. The FN rate was not considered when determining the HFA testing reliability, as previously established^54^. Patients were excluded from the study if they had ocular disease other than glaucoma. When both eyes met all eligibility criteria, the study eye was chosen at random. Enrollment was set at 90 eyes from 90 subjects on the basis of the power calculations described below.

### Size modulation standard automated perimetry

The Octopus 600 perimeter consists of a thin film transistor LCD monitor. Although conventional Octopus SAP can present stimuli to a maximum stimulus intensity level of 4,000 apostilb, the Octopus 600 cannot present stimuli with intensity levels from approximately 400 (10 dB) to 15 (24 dB) apostilb because of limitations of the LCD monitor. Therefore, the stimulus size of high-intensity stimuli more than 10 dB is increased to maintain a stimulus intensity of 10 dB, and the size of low-intensity stimuli less than 24 dB is decreased to maintain an intensity of 24 dB^13^,^14^, according to the following equation:


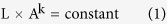


where L is the stimulus luminance, A is the stimulus area, and k is the constant which defines spatial summation. For example, considering the maximum stimulus intensity corresponding to 0 dB with conventional Octopus SAP is 4,000 apostilb,


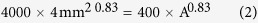


and A is calculated as approximately 64.1 mm^2^. Although the k value used in Goldmann kinetic perimetry is a constant of 0.83, the k value in Octopus 600 SM-SAP is different for each test point. The k values for each test point varied from approximately 0.5 to 1.1 in a previous study for normal and glaucoma patients^13^. Then, a stimulus size of 0 dB with a k value of 0.7 and 1.1 is calculated as approximately 138.1 mm^2^ (between Goldmann size V and VI) and 23.1 mm^2^ (between Golgmann size IV and V), respectively, and that of 35 dB with a k value of 0.5 and 0.8 is calculated as approximately 0.06 mm^2^ (close to Goldmann size 0) and 0.18 mm^2^ (close to Goldmann size II), respectively. In addition, stimulus sizes with SM-SAP of 1 to 9 dB and 25 to 34 dB were calculated using equation (1) and the k value for each test point^13^. The background luminance, stimulus presentation time, testing algorithm, and test point patterns used with the Octopus 600 corresponded to those used with the Octopus 311 and Octopus 900 perimeters. However, the Octopus 600 examination conditions differ from those of C-SAP performed with the HFA. The testing conditions of the Octopus 600 and HFA used in the current study are summarized in Table 3. The perimeter utilizes the novel ‘size modulation’ technique for SAP, which maintains the spatial summation of the stimulus within a dynamic range of 0–35 dB^14^. Inside the Octopus 600 perimeter, the thin film transistor LCD monitor is placed at a distance of 30 cm from the instrument’s eyepiece. All SM-SAP 24-2 Dynamic testing was performed on the study eye in a dark room. Before testing, the participants were required to have their distance refractive error corrected. This was achieved using the built-in 3.25 diopter corrective lens for far distance in the instrument’s eyepiece. The refractive error of subjects with a spherical error between +4.00 and −8.00 diopters and a cylindrical error less than −2.00 diopters was corrected by inserting trial lenses with the spherical equivalent correction into the eye piece. However, subjects with more severe spherical (higher than +4.00 and −8.00 diopters) and cylindrical (higher than −2.00 diopter) errors were corrected with trial frames. The Octopus 600 automatically monitors subject fixation with pupil tracking, but this could not be performed when trial frames were worn by the subject. In these cases, the examiner manually monitored subject fixation on the display monitor throughout the testing. The presentation ratios of FP and FN responses were configured to 10% of the total number of stimuli presented for Octopus 600 testing reliability, which correspond with those of HFA C-SAP performed with the SITA-Standard protocol.

### Visual field testing modality comparisons

Main outcome measures of the current study were how well each global index parameter of SM-SAP and C-SAP correlated. The fovea threshold, mean deviation, and PSD obtained with C-SAP corresponded to fovea threshold, mean defect, and sLV obtained with SM-SAP, respectively^53^.

**Figure 5:**
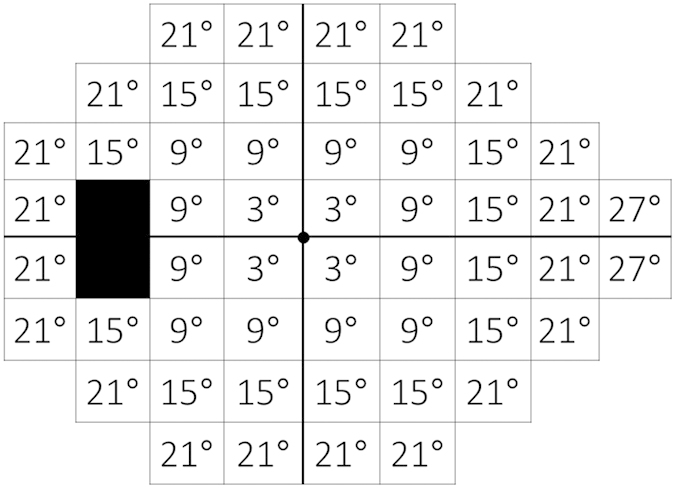
Analysis area dividing each test point of the 24-2 program into five zones. Zones were macula-centered circles with eccentricities of 3°, 9°, 15°, 21°, and 27°^44^,^58^.

The point-wise threshold values, visual field defect size and depth, test duration, and reliability indices (including FP and FN rates) of the two testing modalities were also compared. As in previous studies^44^,^58^, the point-wise threshold values were examined within the central 3°, 9°, 15°, 21°, and 27° zones (Fig. 5). The comparison values obtained with SM-SAP and the TD value obtained with C-SAP were used to compare point-wise and zone threshold values. The Octopus 600 perimeter report displays a ‘+’ when the threshold for that point is within age-corrected normal limits. Comparison values were then calculated using raw data exported by data management software (EyeSuite; Haag-Streit, Koeniz, Switzerland). Defect sizes were compared using the total number of abnormal points, with an abnormality defined as a P value of <5% on the corrected probability plots obtained with SM-SAP and pattern deviation probability plots obtained with C-SAP. Defect depths were compared using the average of the SM-SAP comparison value and the C-SAP TD value for abnormal points identified in corrected probability plots and pattern deviation plots, as was done previously^45^,^46^. For the point-wise threshold value, each test point obtained from the left eye was evaluated as a mirror image so that data from both the left and right eyes could be used together in the analyses. The two test points near the Mariotte blind spot (X, Y = −15°, 3° and −15°, −3°) were excluded from the analyses.

To match HFA reliability criteria, Octopus 600 SM-SAP data were excluded from the analyses if the examiner detected fixation loss on the display or if the FP rate was >15%. The FN rate was not considered in determining SM-SAP testing reliability because the HFA does not calculate FN or pattern deviation for subjects with advanced-stage glaucoma^54^. Therefore, the FN rate, defect size, and defect depth could only be analyzed in subjects for which the HFA calculated these parameters. Decreased sensitivity from age-corrected normative values on SM-SAP performed with the Octopus 600 is represented as a positive value on the report. Therefore, the sign of the SM-SAP sensitivity values was reversed so that the data could be compared to the TD and mean deviation obtained with HFA C-SAP.

### Statistical analyses

Octopus SM-SAP data and HFA C-SAP data were exported as ‘.csv’ files using EyeSuite (Haag-Streit, Koeniz, Switzerland) and HfaFiles (Beeline, Tokyo, Japan) data management software, respectively. All data were analyzed using R statistical software^59^ and G*Power3 (version 3.1.7, Franz Faul, Universität Kiel, Germany). Data normality was evaluated using the Shapiro–Wilk test. Pearson product–moment correlation coefficients (r) and Bland–Altman analysis were used to compare SM-SAP parameters with the corresponding C-SAP parameters. Additionally, the equivalence of a correlation coefficient was analyzed using a χ^2^ test by transforming the correlation coefficients into z-values. Paired *t*-tests or Wilcoxon signed-rank tests were used to compare differences between two dependent means. Statistical significance was defined as P < 0.05.

Using the point biserial model correlation, the effect size, α error, and power (1-β error) were determined to be 0.30, 0.05, and 0.80, respectively, using a two-tailed test. The required sample size was determined to be 82 eyes. Using the difference of two dependent means (matched pairs), the effect size, α error, and power (1-β error) were determined to be 0.50, 0.05, and 0.80, respectively, using a two-tailed test. The required sample size was determined to be 34 eyes.

## Additional Information

**How to cite this article**: Hirasawa, K. *et al.* Comparison of size modulation and conventional standard automated perimetry with the 24-2 test protocol in glaucoma patients. *Sci. Rep.* **6**, 25563; doi: 10.1038/srep25563 (2016).

## Figures and Tables

**Table 1:** Subject demographic and ocular characteristics.

	Mean ± standard deviation	Range
n (eyes) (right/left)	88 (44/44)	
Gender (male/female)	41/47	
Type of glaucoma (eyes)		
Primary open-angle glaucoma	3.9	
Normal-tension glaucoma	30	
Secondary glaucoma	12	
Pre-perimetric glaucoma	3	
Primary closed-angle glaucoma	4	
Age (years)	63.1 ± 13.0	21 to 85
Visual acuity (logMAR)	−0.06 ± 0.11	−0.30 to 0.30
Refraction (diopters)		
Spherical power	−2.20 ± 2.87	−10.75 to 3.75
Astigmatic power	−0.99 ± 0.80	−3.00 to 0.00
Spherical equivalent	−2.69 ± 2.89	−11.13 to 2.75
Intraocular pressure (mmHg)	14.8 ± 3.2	9 to 22

log MAR, logarithm of the minimum angle of resolution.

**Table 2:** Comparison of each parameter measured with size moderation standard automated perimetry (SM-SAP) and conventional SAP (C-SAP).

	SM-SAP	C-SAP	P value
Global indices			
Fovea threshold (dB)	27.6 ± 5.6	34.8 ± 3.1	<0.001*
Mean defect and deviation (dB)	−10.2 ± 6.7	−10.4 ± 8.4	0.306*
sLV and PSD (dB)	7.3 ± 2.4	8.9 ± 4.1	<0.001*
Test duration (seconds)	316.2 ± 51.7	373.0 ± 63.1	<0.001*
Pupil diameter (mm)	4.4 ± 1.2	4.0 ± 1.2	0.030*
Reliability indices			
Fixation loss rate (%)	NA	0 (0–6.8)	NA
False-positive rate (%)	0 (0–0)	1.0 (0–3.0)	0.002†
False-negative rate (%)	7.1 (0–17.9)	1.5 (0–6.0)	<0.001†
Visual field defect size and depth			
Defect size (points)	18.5 ± 9.4	21.8 ± 10.1	0.039*
Defect depth (dB)	−16.4 ± 5.4	−14.3 ± 7.2	0.043*

Data are expressed as mean ± standard deviation or median (inter quantile range).

* and † were analyzed using the paired *t*-test and Wilcoxon signed-rank test, respectively.

sLV, square loss variance; PSD, pattern standard deviation; NA, not available.

**Table 3:** Examination conditions of size modulation standard automated perimetry (SM-SAP) with the Octopus 600 and conventional standard automated perimetry (C-SAP) with the Humphrey field analyzer (HFA).

	SM-SAPOctopus 600	C-SAPHFA
Background luminance	31.4 apostilb	31.4 apostilb
Maximum stimulus intensity	417 apostilb	10,000 apostilb
Stimulus presentation time	0.1 seconds	0.2 seconds
Stimulus size (visual angle)	Goldmann I to VI	Goldmann III
	(0.11° to 3.44°)	(0.43°)
Test point pattern	24-2 (6° interval)	24-2 (6° interval)
Algorithm	Dynamic	SITA-Standard
Fovea threshold	On	On

SITA, Swedish interactive threshold algorithm.
